# Rumen and fecal microbiota profiles associated with immunity of young and adult goats

**DOI:** 10.3389/fimmu.2022.978402

**Published:** 2022-09-13

**Authors:** Tao Luo, Yongtao Li, Wenying Zhang, Jianxin Liu, Hengbo Shi

**Affiliations:** ^1^ Institute of Dairy Science, College of Animal Sciences, Zhejiang University, Hangzhou, China; ^2^ Key Laboratory of Molecular Animal Nutrition (Zhejiang University), Ministry of Education, Hangzhou, China

**Keywords:** microbiota, rumen, feces, young livestock, immune

## Abstract

Low immunity at birth increases risk of disease of young livestock, such as goat kids. Microbiomes change as animals mature, and a healthy microbiome is related to decreased risk of disease. The relationship between microbiota profiles and immunity at different developmental stages remains unclear. Young (female, n = 12, 30 d) and adult (female, n = 12, 2 yrs. old) Saanen dairy goats were used to investigate changes in rumen microbiomes, fecal microbiomes, and their correlations to circulating immune factors. Serum IgG (*P* = 0.02) and IgM (*P* < 0.01) were higher at 2 years than 30 d of age, but there were no differences in IgA (*P* = 0.34), IL-2 (*P* = 0.05), IL-4 (*P* = 0.37) and IL-6 (*P* = 0.73) between ages. Amplicon sequencing analysis revealed young goats had a higher diversity of bacterial communities in rumen and lower diversity in feces compared with adult goats. Ten genera in rumen and 14 genera in feces were positively correlated with serum IgM concentration across both ages. *Olsenella*, *Methanosphaera, Quinella*, *Candidatus_Saccharimonas*, and *Methanobrevibacte*r in rumen and *Ruminobacter*, *Treponema*, *Rikenelaceae_* RC9_ gut_ Group in feces were positively correlated with the concentration of IgG. The correlation analysis using weighted gene co-expression network analysis showed the MEblue module was positively associated with the IgG and IgM. These data provide novel insight into the association between rumen-feces microbiota and immune response. Further experiments are needed to investigate whether inoculating young livestock with immune-related bacteria identified can improve the immune status. Our data suggest a possible strategy to improve the immunity of the kids by alterative microbiota profiles.

## Introduction

Goat kids are born with immature immune systems, making them highly susceptible to diarrhea, inflammation, or pathogenic microbial infections (1–3). Disease incidence decreases growth performance, feed efficiency, and survival rates of livestock. Improving the immunity of young animals would improve their survival rate, welfare, and economic performance as production animals. The role of the gut or rumen microbiome in maintaining health in farm livestock has been gaining increasing interest (4–6). The crosstalk between gut microbe and the host affects animal physiology, metabolism, and immunity (7, 8). A healthy-balanced gut-microbiome is particularly important to the growth, development and metabolism of ruminants, as the microorganisms that occupy the rumen act as an organ that digest fiber to release nutrients that affect immune system development (6, 9).

The fibrolytic activity of rumen microorganisms facilitates the conversion of plant fibers to short-chain fatty acids (SCFA). The SCFA in turn serve as nutrients and are immunomodulatory factors in the host animal (9, 10). The microbiota is composed of microorganisms that include bacteria, archaea, fungi and viruses, for example, *E.coli* and *Helicobacter* (11), *Methanobrevibacter smithii* (12) or *Candida albicans* (13). Homeostasis between commensal and pathogenic organisms is essential to health (14). An imbalance in the composition of microorganisms in the gut, known as dysbiosis, is related to the occurrence of various diseases in both young and adult livestock (15–17). Recent data indicate enrichment of *Proteobacteria* and *Succinivibrionaceae* family in cows is related to low somatic count cells, suggesting that specific rumen microbes can affect the health of the mammary gland (14). Evidence in goats suggests that monitoring of ruminal microbiota or probiotics improves the health of goats, suggesting a relationship between microbiota and immunity (8, 18–20).

In recent years, progress has been made in the application of probiotics to modulate the microbial composition of the gastrointestinal tract to improve the health and immunity of young livestock (7, 8, 21). Our recent studies showed that feeding *Bacillus amyloliquefaciens*-9 to young goats kids increased serum immune factors and decreased the rate of diarrhea, indicating that modification of microbial composition in the gastrointestinal tract improves the health status of kids (20). However, the feeding of microorganisms to change microbiome of the gut has a low efficacy as the rate of colonization is low, and administration by gavage increases the risk of intestinal microbiota disorders.

Dynamic changes occur in bacterial communities in the rumen and the feces of ruminants as they mature (9, 22). Since immunity increases in parallel to changes in gut microbiota, it may be assumed that the microbiota mediates some of these changes in host immunity. Therefore, a potential strategy to improve the immunity of goat kids is through modifying gut microbiome to be more adult-like. Knowledge of the composition difference between the bacterial community in kids and mature animals may help in the identification of potential probiotics with immunomodulatory properties. Further, administration of these potential probiotics to young animals would increase the efficacy of treatment and develop targeted outcomes. In the current study, Saanen dairy goats were sampled at young (30 d of age) and adult (2 yrs. old) age. Serum immune factors and the microbial composition of the rumen and feces were measured and the relationship between circulating immune factors and microbiome was analyzed.

## Materials and methods

### Animals and experiment design

This study was carried out following the regulations of Instructive Notions with Respect to Caring for Experimental Animals and following review and approval of the protocol (protocol no. 201809008) by the Experimental Animal Management Committee of the Zhejiang Sci-technology University.

The twelve young Saanen dairy goats (female, 30 ± 2 d, bodyweight = 6.5 ± 1.05kg) and 12 adult Saanen dairy goats (2 yrs. old, 110 ± 8 d in milk, bodyweight = 52 ± 3 kg, bred at about twenty-month old) were selected for the study from the herd at Baoyuan Dairy Farm (Hangzhou, China). Goats with histories of disease were not included in the experiment. All the animals were collected from one farm to unite the same management and environment. The adults were fed in a block. No kid was included the descendent of any of the old ones. Young kids were born in the shed and stayed with their dam until they were 7 d of age. After separation from dams, each kid was raised in individual nursery in a feeding room at 25 °C and was fed pasteurized whole goat milk twice daily at 08:00 and 15:00 and given free access to the alfalfa hay until 30 ± 2 d. Adult goats were fed with total mixed ration (TMR) diet. The nutrient compositions of the TMR diet are listed in **Supplementary Table**. The adult goats were milked twice daily at 07:30 and 16:00. They were fed twice daily after the lactation at 08:00 and 16:30 and given free access to drinking water.

### Sampling and analysis

Prior to sampling, the ground and walls of the building were treated with insect repellent and disinfected with Bromo Germaine (CAS: 7281-04-1, China National Medicines Corporation Ltd., Beijing, China). The clinical status of animals was evaluated and recorded, and all animals were healthy at both samplings with no signs of diarrhea. The collection of rumen fluid and feces was performed according to the methods described by Fan et al. (23), Wang et al. (24), and Liao et al. (25). Briefly, flexible PVC tube (2 mm of wall thickness × 6 mm of internal diameter) with holes of 2.5 mm diameter in the 15 cm-probe head (Anscitech Co. Ltd. Wuhan, China) was connected to an electric vacuum pump (7 mbar) and was inserted into the rumen of goats *via* the esophagus to collect the rumen sample. About 25 mL of rumen fluid from each goat was collected 3 h after morning feeding using oral stomach tubes. The first 5mL of rumen fluid in each sampling was discarded to remove the potential saliva contamination and the remaining contents was filtered through four layers of cheesecloth. The fecal samples were collected through the rectum stimulus. The rumen and fecal samples were snap frozen in liquid nitrogen until DNA isolation and analysis. The collected samples were divided into four groups: rumen fluid of young (immature rumen, IR) and adult (mature rumen, MR) goats, feces of young (immature cecum, IC) and adult (mature cecum, MC) goats.

Three mL of blood was collected from the jugular vein of each goat before the morning feeding. The rumen fluid and fecal samples were collected and finished within 3 h after the morning feeding at the same day. Serum was separated by centrifugation at 1500 × g for 15 min, transferred to microfuge tubes and stored at -80°C until analysis. Serum immunoglobulin A (IgA, H108-1-2), immunoglobulin G (IgG, H106), immunoglobulin M (IgM, H109), interleukin-2 (IL-2, H003), interleukin-4 (IL-4, H005), and interleukin-6 (IL-6, H007-1-1) were measured using commercial ELISA kits (Nanjing Jiancheng Biotech, Jiangsu, China) according to the manufacturer’s protocol at a wavelength of 450 nm using a micro-titer plate reader (BioTek, USA) (26, 27).

### 16S rRNA gene sequencing

Total genomic DNA of the fecal samples and rumen fluid was extracted using a commercial kit (DP328, Tiangen Biotech, Beijing, China). Briefly, about 1 g (wet weight) of homogenized sample was used for total genomic DNA extraction. The bacteria were lysed by mixing 1 cm and 3 cm beads. The DNA concentration was monitored on 1% agarose gels and was quantified using a Nanodrop 2000 spectrophotometer (Thermo Fisher Scientific, Wilmington, DE, U.S.A). The DNA was amplified using the 341F/806R primer set (341 F: 5′-CCTATYGGGRBGCASCAG-3′, 806R: 5′-GGACTACNNGGGTATCTAAT-3′), which targets the V3-V4 region of the bacterial 16S rRNA gene. Paired-end sequencing (2 × 300 bp) was performed on the Illumina NovaSeq 6000 platform according to the standard protocols (Novogene Technology Co. Ltd., Tianjin, China) (23–25). The identified sequences were deposited in the NCBI Sequence Read Archive (SRA) under the accession No. PRJNA800596.

### Processing of sequencing data

According to the method previously described (28), raw reads of different samples were demultiplexed and quality-filtered to obtain effective tags. QIIME2 (http://qiime.org) was used for bioinformatics analysis. The sequencing data of MR and IR were compared to analyze the differences of rumen microbiome between adult and young goats, and the sequencing data of MC and IC were compared to analyze the differences of fecal microbiome between adult and young goats. The Shannon, Chao1, and Simpson indices were used to estimate the microbial richness and community diversity (29). Principal co-ordinate analysis (PCoA) was applied to assess the dissimilarity of microbial communities between different samples. Linear discriminant analysis Effect Size (LEfSe) was applied to determine differential abundance of bacterial taxa between different samples. The PCoA and LEfSe analysis were performed using the Novomagic (https://magic.novogene.com).

### Statistical analysis

Statistical analysis of serum immune factors and microbial diversity was performed by unpaired t tests. Data were presented as mean ± SEM. Statistical charts were drawn by GraphPad Prism (Version 8.2.1). *P* < 0.05 was considered statistically significant. The Spearman correlation was performed using SPSS software (SPSS v.19, SPSS Inc., Chicago, IL, USA) to explore the relationship among immune factors and bacterial taxa. Correlation heatmaps were generated using the R program pheatmap package. The significant correlation between bacterial genus and the immune globulins and cytokines was considered when |R| > 0.4 and *P* < 0.05. The weighted gene co-expression network analysis (WGCNA) package in R (Version 4.0.2) was used to investigate the relationship between immune indices and microbiome with the soft-thresholding power at 12. The different colors were used to identify different modules. The relationship of microbial composition of positive correlation modules in WGCNA results was further explored, and the network was drawn by Cytoscape (Version 3.8.0).

## Results

### Young goats had a lower concentration of lgG and lgM compared with adults

**Figure 1:**
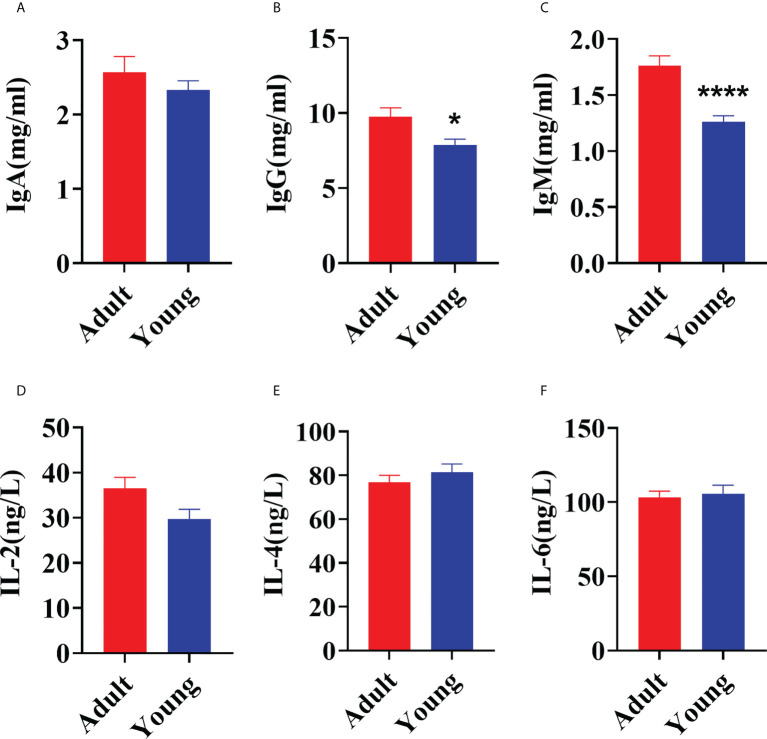
Comparison of serum immune factors concentrations between young (30 ± 2 d) and adult (2 yrs old) goats. **(A)** The concentration of IgA. **(B)** The concentration of IgG. **(C)** The concentration of IgM. **(D)** The concentration of IL-2. **(E)** The concentration of IL-4. **(F)** The concentration of IL-6. The data were analyzed with unpaired t tests, and the data were expressed as mean ± SEM. *P < 0.05 was statistically significance. ****P < 0.0001 was the extremely significance.

Age of the animal significantly affected concentrations of IgG (*P* = 0.02) and IgM (*P* < 0.01) with levels lower when goats were 30 d versus 2 yrs of age (**Figures 1B, C**). There was no difference in serum concentrations of IgA (*P* = 0.34), IL-2 (*P* = 0.05), IL-4 (*P* = 0.37) and IL-6 (*P* = 0.73) between 30 d and 2 yrs of age (**Figures 1A, D–F**).

### Comparison of rumen microbiota profiling between young and adult goats

**Figure 2:**
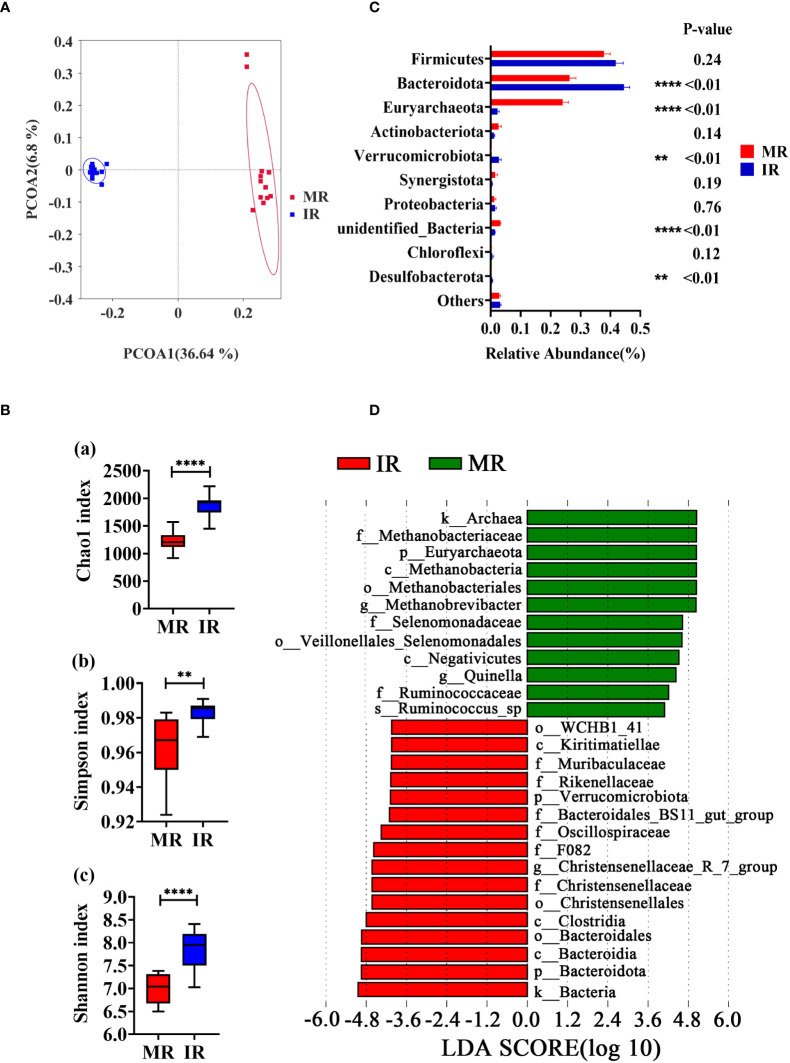
Comparison in rumen microbiome between adults and young goats. **(A)** Principal coordinate analysis (PCoA) of microbial based on unweighted UniFrac distances. **(B)** Alpha diversity (Chao1 index; Simpson index; Shannon index). **(C)** Relative abundance of top10 phyla. **(D)** Bar chart shows LDA score of young and adult goats. LDA score > 4. MR: rumen of adult goats. IR: rumen of young goats. The data were analyzed with unpaired t tests, and the data were expressed as mean ± SEM. **P < 0.01, ****P < 0.0001.

The amplicon sequencing of the 16S rRNA gene in rumen samples found 4677 Operational Taxonomic Units (OTU, **Supplementary File**). The MR and IR group shared 1466 OTUs, and 1194 and 2017 OTUs were uniquely detected, respectively (**Supplementary Figure 1A**). The rarefaction curves (**Supplementary Figure 1B**) gradually leveled off, indicating an even distribution of species and a reasonable amount of sequencing data progressively, allowing for subsequent analysis. Principal co-ordinate analysis (PCoA) based on unweighted UniFrac distances of OTUs showed distinct clustering of samples by age-stage of development (PCoA1 = 36.64%, PCoA2 = 6.8%, **Figure 2A**). Similar data was confirmed by PCoA result based on weighted UniFrac distances (**Supplementary Figure 1C**). Compared with MR group, the index of Chao1 (*P* < 0.01), Simpson (*P* < 0.01), and Shannon (*P* < 0.01) were significantly higher in IR group, indicating a greater diversity of microbial species in the immature compared with mature rumen (**Figure 2B**). According to the data at the phylum level, the dominant phyla of MR and IR were *Firmicutes*, *Bacteroidota*, and *Euryarchaeota*. As shown in **Figure 2C**, the abundances of *Bacteroidota* (*P* < 0.01), *Verrucomicrobiota* (*P* < 0.01), and *Desulfobacterota* (*P* < 0.01) in IR group were higher compared with MR group. Lower levels of *Euryarchaeota* (*P* < 0.01) and *unidentified_Bacteria* (*P* < 0.01) were also observed in the IR group compared with MR. Linear discriminant analysis Effect Size (LEfSe) was used to determine differential abundance of bacterial taxa between MR and IR ages with LDA Score > 4. *Methanobrevibacter* and *Quinella* were enriched in MR whereas the genera *Christensenellaceae_*R-7_group was enriched in the IR group (**Figure 2D**).

### Comparison of fecal microbiota profiles between young and adult goats

**Figure 3:**
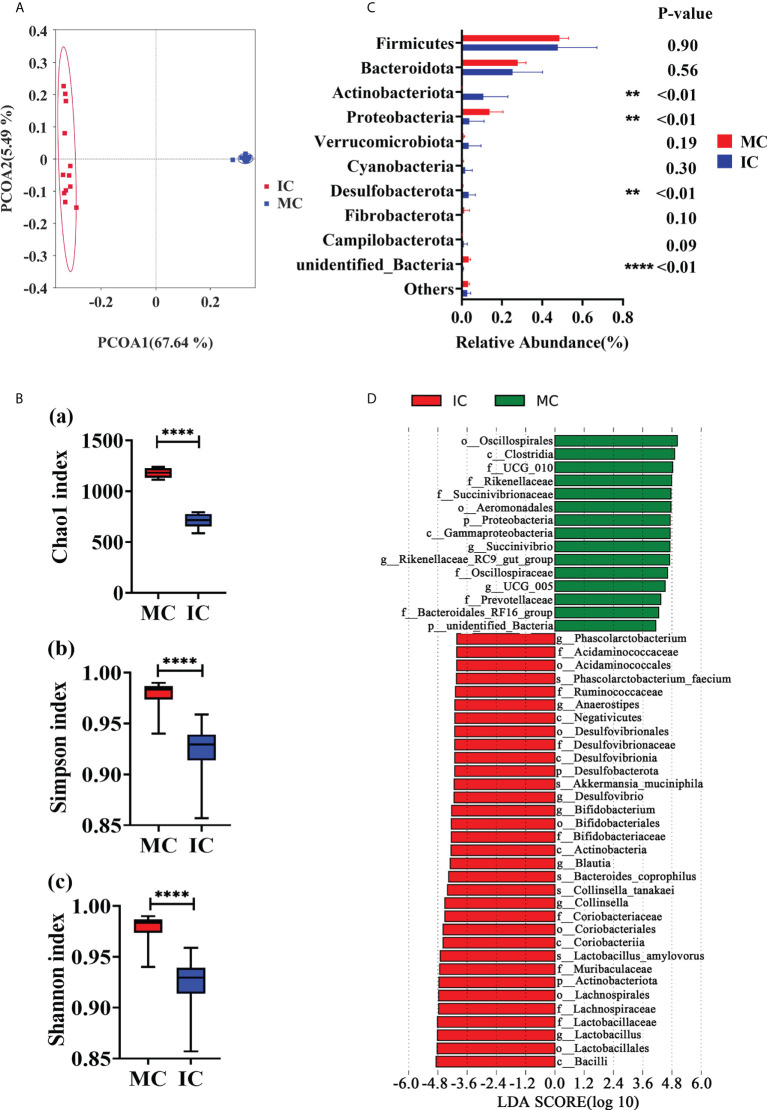
Comparison in fecal microbiome between adults and young goats. **(A)** Principal coordinate analysis (PCoA) of microbial based on unweighted UniFrac distances. **(B)** Alpha diversity (Chao1 index; Simpson index; Shannon index). **(C)** Relative abundance of top10 phyla. **(D)** Bar chart shows LDA score of young and adult groups. LDA score > 4. MC: feces of adult goats. IC: feces of young goats. The data were analyzed with unpaired t tests, and the data were expressed as mean ± SEM. **P < 0.01, ****P < 0.0001.

The amplicon sequences of the 16S rRNA gene were assigned to 2803 OTUs across all the fecal samples (**Supplementary File**), and the data quality was supported by the rarefaction curves (**Supplementary Figure 1E**). The MC and IC group shared 443 OTUs, and 1257 and 1103 OTUs were uniquely detected, respectively (**Supplementary Figure 1D**). Principal co-ordinate analysis (PCoA) based on unweighted UniFrac distances (**Figure 3A**) and weighted UniFrac distances (**Supplementary Figure 1F**) of OTUs showed two distinct clusters by age of the animals. Compared with IC group, the indices of Chao1 (*P* < 0.01), Simpson (*P* < 0.01) and Shannon (*P* < 0.01) were significantly higher in MC group (**Figure 3B**). According to the data at the phylum level, the dominant phyla of MC and IC were *Firmicutes, Bacteroidota, Proteobacteria*, and *Actinobacteriata*. As shown in **Figure 3C**, compared with MC group, the abundances of *Actinobacteriota* (*P* < 0.01) and *Desulfobacterota* (*P* < 0.01) in IC group were higher. Lower levels of *Proteobacteria* (*P* < 0.01) and *unidentified_Bacteria* (*P* < 0.01) were also observed in the IC group compared with MC. The most differentially abundant bacterial taxa in the MC group tested by the LEfSe analysis (LDA Score > 4) belonged to the genera *Succinivibrio*, *Rikenellaceae_*RC9_gut_group, and UCG_005. The genera *Phascolarctobacterium*, *Anaerostipes*, *Desulfovibrio*, *Bifidobacterium*, *Blautia*, *Collinsella*, and *Lactobacillus* were enriched in the IC group (**Figure 3D**).

### Comparison of microbiota profiles between rumen and feces

**Figure 4:**
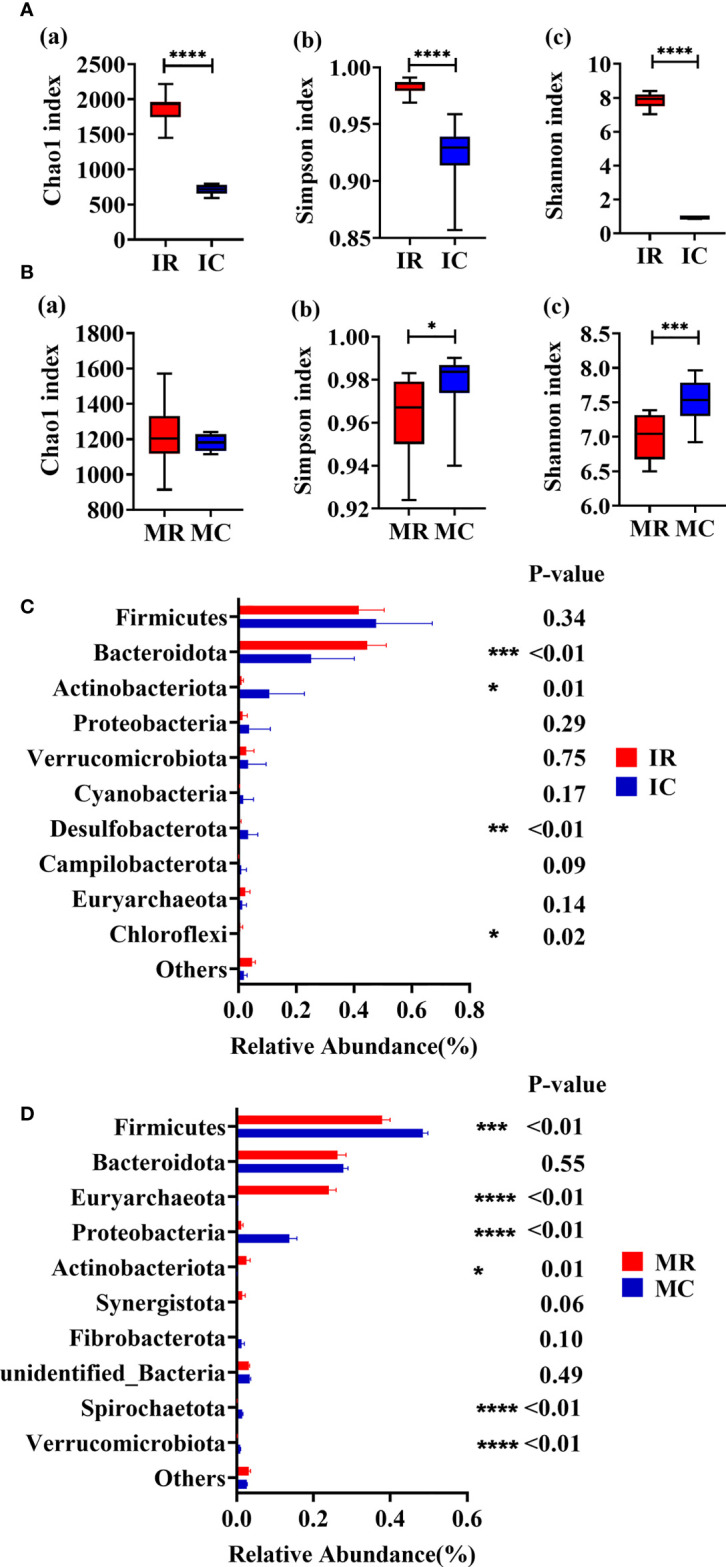
Rumen-feces microbial composition fluctuates with age. **(A)** Alpha diversity of young (Chao1 index; Simpson index; Shannon index). **(B)** Alpha diversity of adult (Chao1 index; Simpson index; Shannon index). **(C)** Relative abundance of top10 phyla of young. **(D)** Relative abundance of top10 phyla of adult. MC: feces of adult goats. IC: feces of young goats. MR: rumen of adult goats. IR: rumen of young goats. The data were analyzed with unpaired t tests, and the data were expressed as mean ± SEM. *P < 0.05, **P < 0.01, ***P < 0.001, ****P < 0.0001.

The microbial compositions between rumen and feces at young and adult age, respectively, were analyzed to assess the fluctuation of microbiota with ages. Venn diagram and PCOA results indicated differences in rumen and fecal microorganisms (**Supplementary Figure 2**). The indices of Chao1 (*P* < 0.01), Simpson (*P* < 0.01) and Shannon (*P* < 0.01) were significantly higher in the IR group compared with IC group (**Figure 4A**). Higher indices of Simpson (*P* = 0.03) and Shannon (*P* < 0.01) were observed in the MC group compared to the MR (**Figure 4B**). Compared with IC group, IR group has higher abundances of *Bacteroidota* (*P* < 0.01) and *Chloroflexi* (*P* = 0.02) and lower abundance of *Actinobacteriota* (*P* = 0.01) and *Desulfobacterota* (*P* < 0.01) (**Figure 4C**). Compared with MC group, lower levels of *Firmicutes* (*P* < 0.01), *Proteobacteria* (*P* < 0.01), *Spirochaetota* (*P* < 0.01) and *Verrucomicrobiota* (*P* < 0.01) and higher levels of *Euryarchaeota* (*P* < 0.01) and *Actinobacteriota* (*P* = 0.01) were also observed in the MR group (**Figure 4D**).

### Rumen and fecal microbiome associated with increasing concentration of immune factors

**Figure 5:**
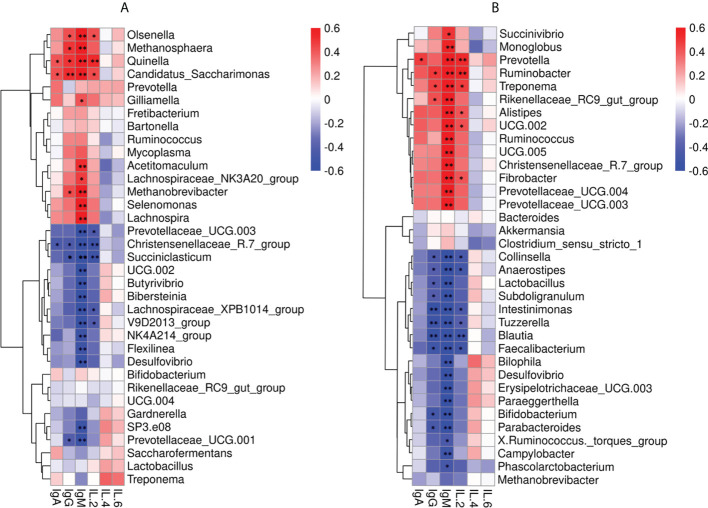
Correlation between the immune factors and the rumen and fecal bacteria at the genus level. **(A)** Spearman correlation between rumen samples (MR, IR) and serum immune factors. **(B)** Spearman correlation between fecal samples (MC, IC) and serum immune factors. *P < 0.05, **P < 0.01.

Spearman correlation analysis was performed to determine whether genera of fecal and rumen bacteria were associated with circulating levels of IgA, IgG, IgM, IL-2, IL-4, and IL-6. Data analysis across both ages found IgM was positively correlated to 10 genera in rumen including *Olsenella* (*P* < 0.01, *R* = 0.73), *Methanosphaera* (*P* < 0.01, *R* = 0.65), *Quinella* (*P* < 0.01, *R* = 0.76), *Candidatus_Saccharimonas* (*P* < 0.01, *R* = 0.72), *Gilliamella* (*P* = 0.04, *R* = 0.42), *Acetitomaculum* (*P* < 0.01, *R* = 0.52), *Lachnospiraceae_*NK3A20_group (*P* = 0.02, *R* = 0.48), *Methanobrevibacter* (*P* < 0.01, *R* = 0.62), *Selenomonas* (*P* < 0.01, *R* = 0.64), and *Lachnospira* (*P* < 0.01, *R* = 0.63). Five rumen bacteria were positively correlated with serum IgG including *Olsenella* (*P* = 0.04, *R* = 0.43), *Methanosphaera* (*P* = 0.02, *R* = 0.49)*, Quinella* (*P* = 0.01, *R* = 0.50), *Candidatus_Saccharimonas* (*P* < 0.01, *R* = 0.52), and *Methanobrevibacte*r (*P* = 0.04, *R* = 0.43). The 13 genera in rumen were negatively correlated with IgM including *Prevotellaceae_*UCG.003 (*P* < 0.01, *R* = -0.62), *Christensenellaceae_*R.7_group (*P* < 0.01, *R* = -0.75), *Succiniclasticum* (*P* < 0.01, *R* = -0.77), UCG.002 (*P* < 0.01, *R* = -0.60), *Butyrivibrio* (*P* < 0.01, *R* = -0.68), *Bibersteinia* (*P* < 0.01, *R* = -0.69), *Lachnospiraceae_*XPB1014_group (*P* < 0.01, *R* = -0.74), V9D2013_group (*P* < 0.01, *R* =- 0.71), NK4A214_group (*P* < 0.01, *R* = -0.53), *Flexilinea* (*P* < 0.01, *R* = -0.53), *Desulfovibrio* (*P <*0.01, *R* = -0.58), SP3.e08 (*P* < 0.01, *R* = -0.55), and *Prevotellaceae_*UCG.001 (*P* < 0.01, *R* = -0.55). Whereas *Christensenellaceae_*R.7_group (*P* = 0.03, *R* = -0.44), *Succiniclasticum* (*P* = 0.02, *R* = -0.47), and *Prevotellaceae_*UCG.001 (*P* = 0.05, *R* = -0.41) were negatively correlated with IgG (**Figure 5A**).

The 14 genera in feces were also positively correlated with IgG including *Succinivibrio* (*P* = 0.01, *R* = 0.50), *Monoglobus* (*P* < 0.01, *R* = 0.56), *Prevotella* (*P* < 0.01, *R* = 0.73), *Ruminobacter* (*P* < 0.01, *R* = 0.78), *Treponema* (*P* < 0.01, *R* = 0.77), *Rikenelaceae_* RC9_ gut_ Group (*P* < 0.01, *R* = 0.68), *Alistipes* (*P* < 0.01, *R* = 0.61), UCG.002 (*P* < 0.01, *R* = 0.71), *Ruminococcus* (*P* < 0.01, *R* = 0.67), UCG.005 (*P* < 0.01, *R* = 0.68), *Christensenellaceae_*R.7_group(*P* < 0.01, *R* = 0.66), *Fibrobacter* (*P* < 0.01, *R* = 0.73), *Prevotellaceae_*UCG.004 (*P* < 0.01, *R* = 0.70), and *Prevotellaceae_*UCG.003 (*P* < 0.01, *R* = 0.66). *Ruminobacter* (*P* = 0.02, *R* = 0.49), *Treponema* (*P* = 0.02, *R* = 0.46), and *Rikenelaceae_*RC9_gut_Group (*P* = 0.04, *R* = 0.42) were positively correlated with IgM. Furthermore, 17 genera in feces were negatively correlated with IgM including *Collinsella* (*P* < 0.01, *R* = -0.73), *Anaerostipes* (*P* < 0.01, *R* = -0.71), *Lactobacillus* (*P* < 0.01, *R* = -0.70), *Subdoligranulum* (*P* < 0.01, *R* = -0.74), *Intestinimonas* (*P* < 0.01, *R* = -0.74), *Tuzzerella* (*P* < 0.01, *R* = -0.71), *Blautia* (*P* < 0.01, *R* = -0.83), *Faecalibacterium* (*P* < 0.01, *R* = -0.78), *Bilophila* (*P* < 0.01, *R* = -0.55), *Desulfovibrio* (*P* < 0.01, *R* = -0.58), *Erysipelotrichaceae_*UCG.003 (*P* < 0.01, *R*= -0.63), *Paraeggerthella* (*P* < 0.01, *R* = -0.63), *Bifidobacterium* (*P* < 0.01, *R* = -0.65), *Parabacteroides* (*P* < 0.01, *R* = -0.66), *X.Ruminococcus._torques_group* (*P* = 0.03, *R* = -0.45), *Campylobacter* (*P* < 0.01, *R* = -0.52), and *Phascolarctobacterium* (*P* = 0.02, *R* = -0.47). Ten genera in feces were negatively correlated with IgG including *Collinsella* (*P* = 0.03, *R* = -0.43), *Anaerostipes* (*P* = 0.05, *R* = -0.41), *Lactobacillus* (*P* = 0.04, *R* = -0.43), *Subdoligranulum* (*P* = 0.04, *R* = -0.42), *Intestinimonas* (*P* < 0.01, R = -0.60), *Tuzzerella* (*P* < 0.01, *R* = -0.53), *Blautia* (*P* < 0.01, *R* = -0.59), *Faecalibacterium* (*P* = 0.01, *R* = -0.51), *Bifidobacterium* (*P* = 0.02, *R* = -0.49), and *Parabacteroides* (*P* = 0.03, *R* = -0.46) (**Figure 5B**).

### Hub microbiota correlated with the immune factors

**Figure 6:**
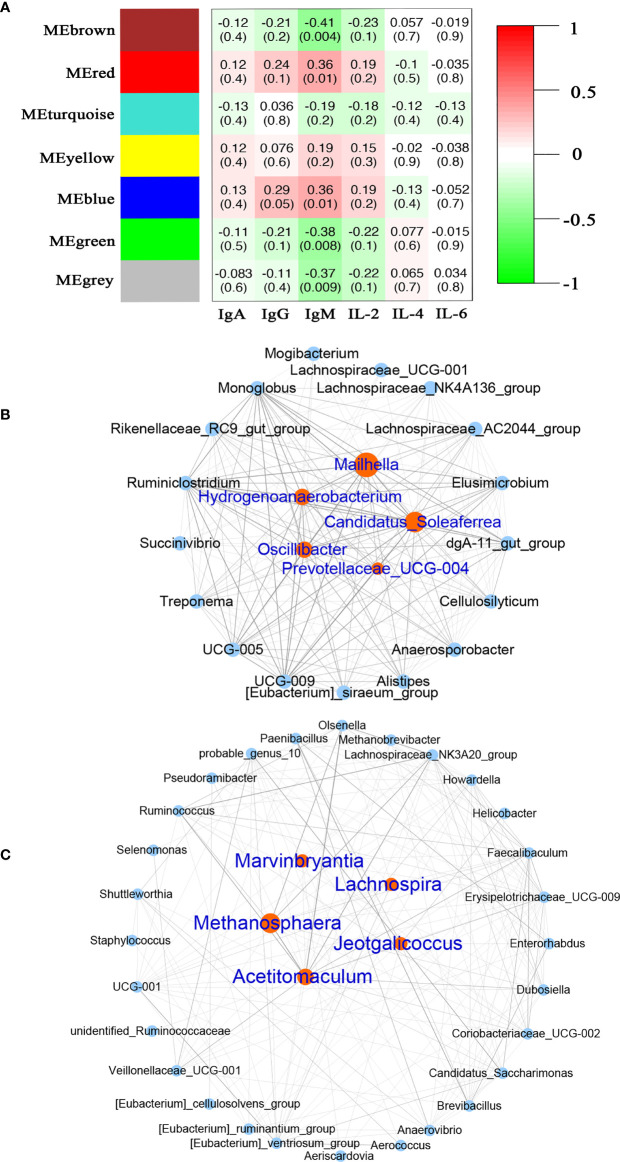
Correlation between the serum immune factors and abundance of microbiome in rumen and feces at the genus level. The correlated module was analyzed using a weighted gene co-expression network analysis (WGCNA). Correlation networks were generated using Spearman’s rank correlation coefficients. **(A)** The heatmap of the WGCNA module. **(B)** Microbiome interaction network and hub microbes in the MEred module. **(C)** Microbiome interaction network and hub microbes in the MEblue module.

At the genus level, we further investigated the relationship between immune indices and microbiome using WGCNA. A total of seven related microbiota modules were identified (**Figure 6A**). MEBlue was significantly correlated with both IgM (*P* = 0.01, *R* = 0.36) and IgG (*P* = 0.05, *R* = 0.29) while the MEred was significantly correlated with IgM (*P* = 0.01, *R* = 0.36). The network exported from the MEred module showed that *Mailhella*, *Candidatus_Soleaferrea*, *Hydrogenoanalobacterium*, *Oscillibacter*, and *Prevotellaceae_*UCG-004 were the hub microbiota in the MEred module (**Figure 6B**). The network derived from the MEblue module showed that that *Methanosphaera*, *Acetitomaculum*, *Marvinbryantia*, *Lachnospira*, and *Jeotgalicoccus* were the hub microbiota in the MEblue module (**Figure 6C**).

## Discussion

Young livestock are highly susceptible to microbial infections due to their immature immune systems, which leads to an unbalanced microbiome (4, 5). In the current study, microbial profiles of rumen and feces of 24 healthy dairy goats were measured and compared between early development and adult stages to identify changes that occur in distribution and their relationship to circulating immunoglobulins. As animals mature, the microbial distribution changes and we found bacterial microbiota abundance in rumen and fecal matter associated with serum IgM and IgG levels.

The immune system of ruminants provides defense against pathogenic microbiome by secreting immunoglobulins (30, 31). The serum immunoglobulin concentrations directly reflect the resistance to exogenous pathogenic microbiota (32). In the current study, the higher concentrations of IgG and IgM in adult goats suggested a mature immune system in adults. No differences between goats at 30 d and 2 yrs were observed for interleukins, agreeing with the fact that interleukin levels in ruminants are sensitive to diseases caused by bacterial infections (e. g., mastitis and diarrhea) and there are no differences between healthy individuals (26, 33, 34).

The diversity of fecal microbiome in adults was higher than that of young, indicating a continuous colonization of intestinal microbiome (35, 36). In particular, the diversity of gut microbes has been proposed as a new marker for assessing gut health and metabolic capacities (37). In agreement with previous reports (38), the dominant phylum among gut microbial community in kids and adults were *Firmicutes* and *Bacteroidota*. The increasing abundance of *Proteobacteria* at 2 yrs old is consistent with the fact that *Proteobacteria* contributed to the host’s energy and nutrient demands (39). The levels of IgM and IgG were strongly associated with the abundance of the microbiota profiles, suggesting the idea that modulating IgM- and IgG-associated microbial community in the gastrointestinal tract of young goats could improve their immunity early in life. The abundance of 14 genera in the feces positively associated with the concentration of IgM supports the idea. Among the 14 genera, at least, *Prevotella* (40) and *Alistipes* (41) are related to inflammation and disease protection. These results suggest that IgM- and IgG-associated microbiota play an important role in maintaining gut health.

Goats at 30 d have been on a milk hay-diet and the rumen is just starting to develop and at 2 yrs old fully functional. Although the process of the microbial construction in the young livestock is still unclear (22, 42), along with the fact that young livestock are immunocompromised, higher diversity in the young rumen suggests that young rumen is more susceptible to the invasion of exogenous microorganisms from food or environment. This is further supported by the instability of the rumen microbiota of young goats (43) and the finding of higher diversity in the rumen microbiota of young goats. Compared with less mature communities, a well-established microbial community within the rumen has higher resilience and is more resistant to disturbances (44–46). Along with the fact that a long-term early life intervention can affect the composition of the rumen microbial community, the rudimentary status of the ruminants’ microbiota in the early life provides a possibility for rumen microbiota modification (44). Consistent with previous data in the rumen of goats and sheep (24, 47), the dominant phylum in kids and adults were *Firmicutes* and *Bacteroidota*, which are associated with carbohydrate and protein metabolism.

Early-life microbial succession in newborn offspring is essential for immune development and may play a crucial role in animal resilience to pathogens later in life (48). This idea is supported by the evidence that probiotics feeding efficiently had a positive effect on diarrhea of young goats (20) and mice (49). The correlation analysis showed that the 10 genera in the rumen strong positive associated with the concentration of IgM. Among the 10 genera, both *Lachnospira* (50, 51) and *Acetitomaculum* (52, 53) belong to the *Lachnospiraceae* family, which is a major producer of SCFA, known to enhance the integrity epithelial barrier and inhibiting inflammation. *Methanosphaera* and *Methanobrevibacter* are typical methanogens, which have been reported to be associated with inflammatory response (54, 55). The dominance of *Lachnospira*, *Acetitomaculum*, and *Methanosphaera* in microbial interactions network in WGCNA data further highlights their importance in the immune response.

The fact that inoculation of the rumen microbiota in the early life stages of goats is an effective strategy to accelerate rumen development (56), suggested that probiotics collected from the rumen may be more accessible to the microbial community than in non-ruminal species. It is supported by the evidence that feeding rumen fluid to newborn lambs significantly improved their feed digestibility and growth performance, and the finding that the presence of adult companion goats interfered with the development of rumen function in young goats (57, 58). The previous data that inoculation of calves with rumen microbiota reduce diarrhea (59) is consistent with the current data that the rumen is an ideal targeted organ to improve the immune response at the young stage for the livestock. However, further research is needed to assess the immune-related bacteria identified in the current study to improve the immunity in young livestock.

A limitation of the present study is realized. Firstly, the function of the immune system is influenced by various factors, including hormones (60). The experimental animals used in this study were all females and the sex hormones may affect the development of the adults and, thus, change the microbiota profiles in rumen or feces. However, adult female goats are the dominant individuals in the farms, and they have a direct interaction with the kids through milk feeding. Although the correlation between bacteria and IgG and IgM parameters is of interest, the interpretation could be problematic or questionable based on 16S sequencing. The measurement of specific antibodies against those bacteria would be required to understand the effects of the observed results.

## Conclusion

It is considered an ideal strategy using endogenic microbiota from the rumen or feces to keep the health status of young livestock. In the current study, we firstly jointly considered both rumen and fecal microbiota and their correlation with immunity response at different development stages. A higher level of IgM and IgG was observed in adult goats. Ten genera in rumen and 14 genera in feces were positively correlated with serum IgM concentration. *Olsenella*, *Methanosphaera, Quinella*, *Candidatus_Saccharimonas*, and *Methanobrevibacte*r in rumen and *Ruminobacter*, *Treponema*, *Rikenelaceae_* RC9_ gut_ Group in feces were positively correlated with the concentration of IgG. Further experiments would be performed to investigate whether the immune-related bacteria identified in the current study would improve the immunity in young livestock. However, our results in the current study improve our understanding of the rumen and fecal microbiota under different ages and their association with immune response. These data suggest a possible strategy to improve the immunity of the kids by alterative microbiota profiles.

## Data availability statement

The datasets presented in this study can be found in online repositories. The names of the repository/repositories and accession number(s) can be found below: https://www.ncbi.nlm.nih.gov/, PRJNA800596.

## Ethics statement

The animal study was reviewed and approved by The Experimental Animal Management Committee of the Zhejiang Sci-technology University.

## Author contributions

TL: data curation, and writing—original draft. YL: software and project administration. WZ: data curation. JL: project administration. HS: funding acquisition and supervision, and writing- editing draft. All authors contributed to the article and approved the submitted version.

## Funding

This study was jointly supported by grants from Key R&D program of Zhejiang Province (2022C04017) and Zhejiang Provincial Major Science and Technology Projects on Agricultural New Varieties Selection and Breeding (2021C02068-6).

## Acknowledgments

The authors thank Dr. Theresa M. Casey from Purdue University for the helpful discussion and language editing. The authors also thank the owners and staff of Baoyuan Dairy Farm (Hangzhou, China) for allowing the use of their lactating and young goats in this experiment.

## Conflict of interest

The authors declare that the research was conducted in the absence of any commercial or financial relationships that could be construed as a potential conflict of interest.

## Publisher’s note

All claims expressed in this article are solely those of the authors and do not necessarily represent those of their affiliated organizations, or those of the publisher, the editors and the reviewers. Any product that may be evaluated in this article, or claim that may be made by its manufacturer, is not guaranteed or endorsed by the publisher.
