# Supplementary figures and images for: Rumen and fecal microbiota profiles associated with immunity of young and adult goats
Source: Front Immunol. 2022 Sep 13;13:978402. doi: 10.3389/fimmu.2022.978402 (PMC9513485; doi:10.3389/fimmu.2022.978402)

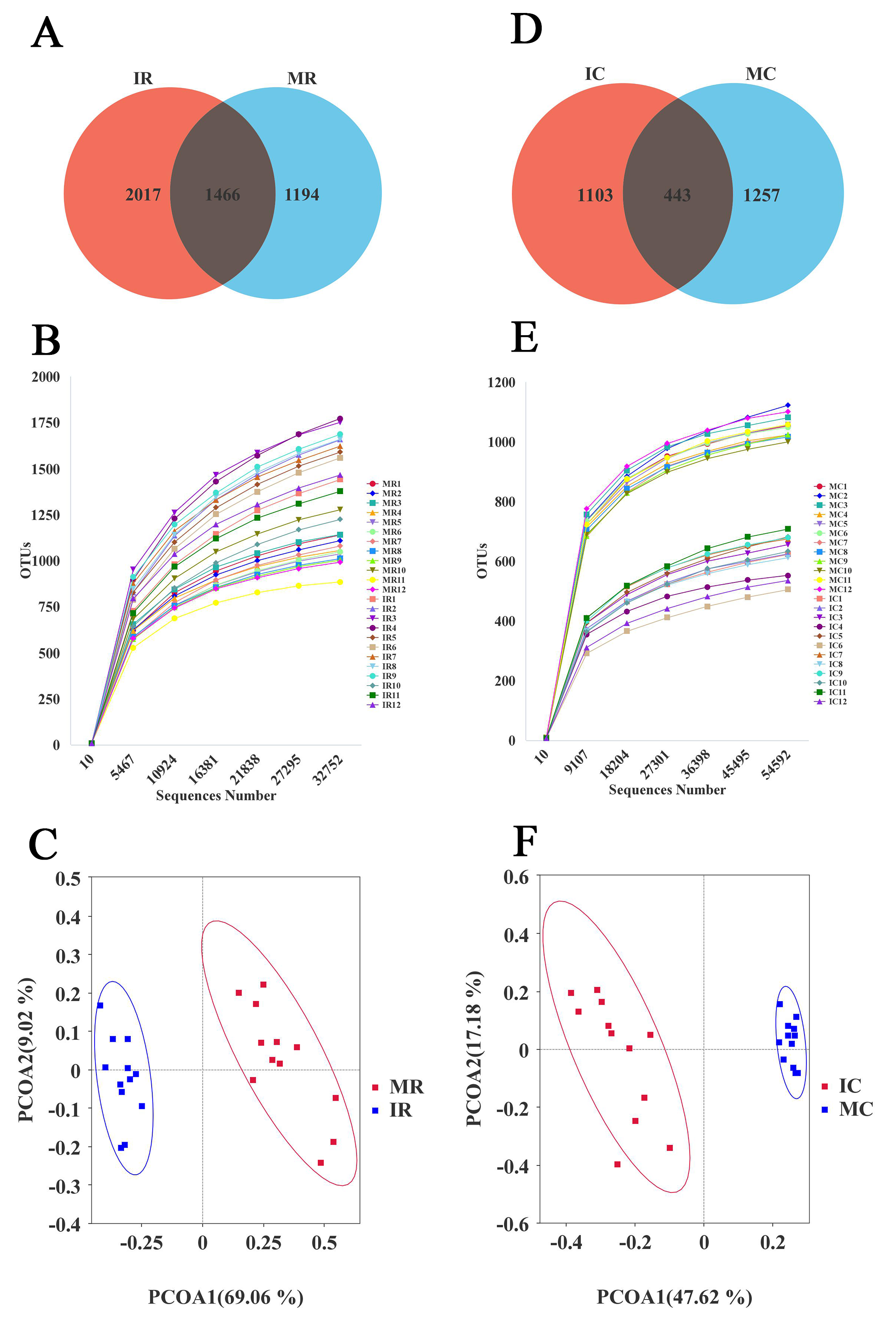

Supplement: Supplementary file 1 [file Image_1.tif]

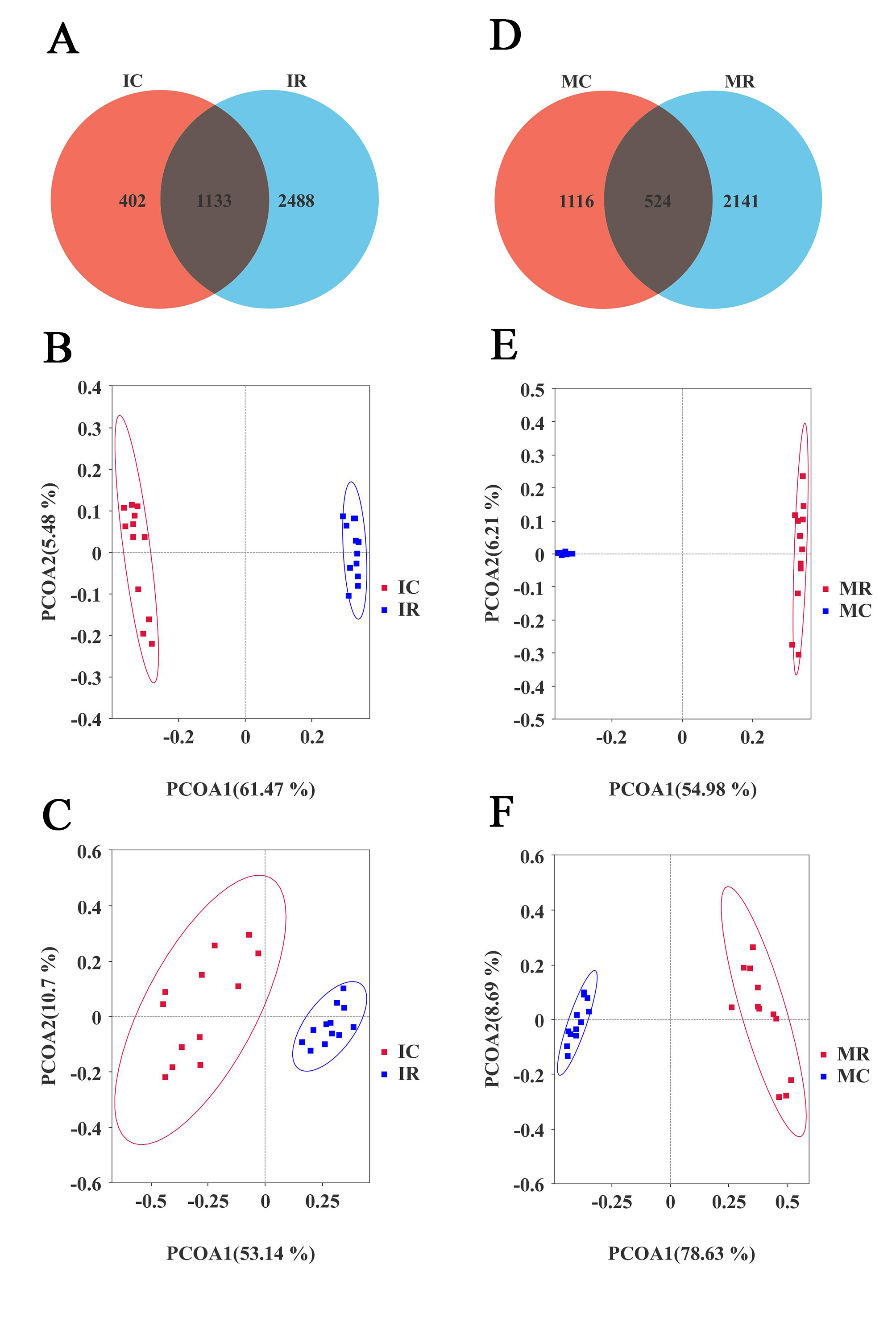

Supplement: Supplementary file 2 [file Image_2.tif]
