# Supplementary material for: Rumen and fecal microbiota profiles associated with immunity of young and adult goats
Source: Front Immunol. 2022 Sep 13;13:978402. doi: 10.3389/fimmu.2022.978402 (PMC9513485; doi:10.3389/fimmu.2022.978402)
Supplement: Supplementary file 4 [file Table_2.docx]

**Supplementary Table. Nutrient compositions of the basal diet.**

| **Items** | **Experimental diets** |
| --- | --- |
| *Ingredient (of DM basis %)* | |
| Whole corn silage | 34.3 |
| Alfalfa hay | 20.1 |
| Corn | 27.8 |
| Wheat bran | 12.6 |
| Soya bean meal | 3.2 |
| Premix | 1.2 |
| NaCl | 0.3 |
| NaHCO_3_ | 0.5 |
| Total | 100 |
| *Nutrient composition* | |
| Dry matter (%) | 47.9 |
| Crude protein (%) | 10.4 |
| Ether extract (%) | 3.4 |
| Crude fibre (%) | 18.5 |
| Neutral detergent fibre (%) | 44.6 |
| Acid detergent fibre (%) | 23.7 |
| Ash (%) | 4.1 |
